# How we help our patients get their medicines and use them safely

**Published:** 2023-05-22

**Authors:** Bitrus Dauda, Hamida Musa, Farouk Garba

**Affiliations:** Manager: Eye Clinic, Hajiya Gambo Sawaba General Hospital, Zaria, Nigeria.; Deputy Sister: Eye Clinic, Hajiya Gambo Sawaba General Hospital, Zaria, Nigeria.; Senior Lecturer/Consultant Ophthalmologist: Department of Ophthalmology, College of Medical Sciences, Ahmadu Bello University, Zaria, Nigeria.

**Setting up a pharmacy inside the eye clinic helped patients get their medicines safely, quickly, and without incurring additional expense.**

**Figure:**
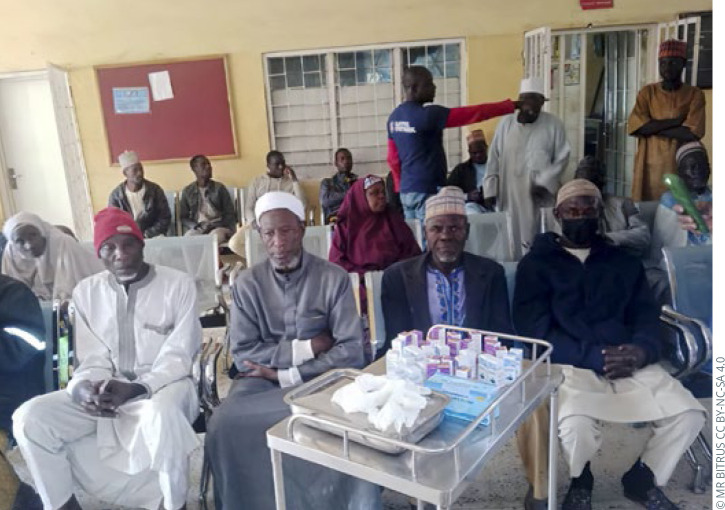
After surgery and a postoperative examination, cataract patients and their care givers gather in a hall where health workers give explanations and answer questions about postoperative care, including how to use their medicines. nigeria

Hajiya Gambo Sawaba General Hospital is a secondary centre in Zaria, in the northern part of Nigeria. The majority of patients who come to the hospital have no medical insurance and have to make out-of-pocket payments for treatment and medication.

Since the hospital pharmacy did not stock eye medicines, eye clinic personnel directed patients to pharmacies in the local area which were known to sell good quality medicines at reasonable prices. Patients were asked to return to the eye clinic afterwards so that the nurses could check they had purchased the correct medication.

However, we noticed that patients coming from small villages had difficulty finding their way around, even inside the hospital, and struggled to find the pharmacies the eye department recommended. We also noticed that patients were spending additional funds to transport themselves in search of medications elsewhere, making the experience overwhelming and expensive.

## A new approach

To address this issue, the eye clinic manager and his team advocated for a pharmacy to be set up right inside the eye clinic itself. The hospital administrators agreed, and the eye clinic pharmacy was set up in November 2021. It is overseen by the main hospital pharmacy and stocks commonly prescribed medicines such as antibiotics, anti-inflammatories, analgesics, and glaucoma medication. Consumables, such as viscoelastic, dilation drops, and blocking agents, are also kept in stock.

Now that the pharmacy is inside the eye clinic, eye patients can easily and quickly get the medication they need. They would only be sent to a local pharmacy if an item was out of stock in the hospital. When this happens, a health worker will first call the pharmacy to ensure that the medicine is available, or find a suitable alternative, before sending the patient to that pharmacy.

This approach assists patients and caregivers greatly by preventing them walking around in an unfamiliar area, looking for a particular medication and perhaps being vulnerable to being exploited or taken advantage of.

## Quality and safety

Hospitals in Nigeria receive medication from the central store in their state. The central store carries out quality control and other checks before accepting medicines from pharmaceutical companies and distributing them to hospitals in the area. The eye clinic pharmacy sends the list of medicines it needs to the hospital pharmacy, which in turn requests these from the state's central store.

Any expired medicines are sent to the hospital pharmacy, which returns it to the central store for safe disposal.

For drug surveillance and adverse drug reactions, the eye clinic pharmacy follows the same protocol as the main pharmacy. Pharmaceutical companies also visit the eye clinic at intervals actively enquire if there have been any problems with their medicines.

“The majority of patients have no medical insurance and resort to out of pocket payment of health services and purchase of medications.”

## The “Show and Teach” system

The ophthalmic nurses in the eye clinic have a system they call “show and teach”. After purchasing medicines, and before leaving, patients are asked to “show” their medicines and prescription to the nurses for verification. This is used as an opportunity to carefully “teach” how the medicines are to be used, and to explain what possible side effects to expect. The date for the patient's next appointment is also confirmed.

